# Fabrication and Photoluminescence Study of Large-Area Ordered and Size-Controlled GeSi Multi-quantum-well Nanopillar Arrays

**DOI:** 10.1186/s11671-016-1312-1

**Published:** 2016-02-24

**Authors:** Yuwen Jiang, Shufan Huang, Zhichao Zhu, Cheng Zeng, Yongliang Fan, Zuimin Jiang

**Affiliations:** State Key Laboratory of Surface Physics, Key Laboratory of Micro and Nano Photonic Structures (Ministry of Education), Collaborative Innovative Center of Advanced Microstructures, Department of Physics, Fudan University, Shanghai, 200433 Peoples’ Republic of China; Shanghai Key Laboratory of Special Artificial Microstructure Materials and Technology, School of Physics Science and Engineering, Tongji University, Shanghai, 200092 Peoples’ Republic of China; Wuhan National Laboratory for Optoelectronic, School of Optoelectronic Science and Engineering, Hua Zhong University of Science and Technology, Wuhan, 430074 Peoples’ Republic of China

**Keywords:** GeSi, Quantum wells, Nanopillars, Photoluminescence, 78.67.-n, 61.72uf, 81.07.St, 62.23.Hj

## Abstract

Large-area ordered GeSi multi-quantum-well nanopillar array (MQW-NPA) samples with different nanopillar lateral sizes (270, 120, and 70 nm) are fabricated by a cost-effective and scalable dry-etching process in combination with nanosphere lithography technique. A significant enhancement in photoluminescence (PL) intensity has been observed in the GeSi MQW-NPA samples compared with the as-grown GeSi MQW one. Nanopillar samples with different lateral sizes show different enhancements in PL intensity. The enhancements are analyzed quantitatively and attributed to three factors. One is the antireflection of the nanopillar structures. Another is an enhanced extraction in nanopillar arrays at the emission wavelength. Thirdly, the GeSi quantum wells in close proximity to the substrates would have more contribution to the PL than before etching. Our results show that all the three factors should be taken into account in designing and fabricating surface microstructures of GeSi MQW materials in order to improve their optical properties.

## Background

During the past decades, ordered semiconductor nanostructures, such as nanowires or nanopillar arrays, have drawn wide attention due to their promising applications in field-effect transistors [1, 2], light-emission devices [3], solar cells [4], novel electronic devices [5], lithium-ion battery [6], and so on. Si-based nanostructures are especially attractive because of their compatibility with current CMOS integrated circuits, however, their optical applications are largely limited due to the nature of their indirect bandgap and low quantum efficiency. Thus, several strategies have been proposed to resolve the low quantum efficiency of Si; among these, one is to integrate Si with other semiconductors like Ge to form heterostructures, such as Ge/Si quantum wells (QWs), quantum dot crystals, and nanowires (NWs) [7–11]. To further improve the quantum efficiency of Si/Ge heterostructures, different surface microstructures have also been proposed. The Choi group [12] observed a huge enhancement in the photoluminescence (PL) spectra from microdisk arrays of Si/Ge/Si SQW and proposed several mechanisms for the enhancement. One is the localization of excitons, which are spatially confined by the diameter of the microdisk. Another is the reduced nonradiative channels in the microdisk-patterned case. At the same time, they also pointed out that the real mechanisms for the PL enhancement were still not clear. The Chen group fabricated a randomly positioned SiGe/Si multi-quantum-well (MQW) nanowall by using electron cyclotron resonance plasma etching on an as-grown SiGe/Si MQW, and the measured electroluminescence intensity of the SiGe/Si MQW nanowalls increased to about 1.5 times for the enhancement of emission light extraction [13]. Chen et al. also observed PL emission enhancement in size-uncontrollable Si/Ge superlattice pyramidal nanodots, which were fabricated by chemical selective etching through a self-assembled Ge QD mask [14]. Recently, Chang et al. successfully fabricated ordered SiGe/Si MQW nanorod and nanodot arrays with different heights by combining nanosphere lithography and reactive ion-etching (RIE) process [15]. However, in the PL spectra, the intensity of the Si/Ge MQW nanorod and nanodot array nanostructure samples did not increase significantly. Meanwhile, a blueshift was observed, which was explained by the QW nonuniformity in stain distribution and Ge composition along the growth direction together with a larger penetration depth of incident light in the nanorod samples. So, it seems that the mechanisms of surface microstructure in improving the PL emission properties of GeSi QWs are still not clear, especially in the quantitative analysis. Few studies have concerned how the lateral sizes of the nanopillar affected the PL enhancement.

In this paper, large-area ordered and size-controlled GeSi MQW nanopillar arrays with different nanopillar lateral sizes were fabricated by a combination of nanosphere lithography and dry etching. Compared to the as-grown GeSi MQWs, considerable enhancements in PL intensity have been observed in the GeSi MQW-NPA samples. The sample with the largest nanopillar size (270 nm) shows the most obvious enhancement. Three factors are analyzed quantitatively or qualitatively to explain the enhancements. One factor is the antireflection of the nanopillar structures. The second one is the light extraction efficiency in nanopillar arrays at the emission wavelength. In addition, the GeSi quantum wells in close proximity to the substrates would have more contribution to the PL than before etching, due to a large penetration depth for the incident light, which is the third factor. In our study, we focus on the quantitative analyses for the PL enhancements in the GeSi multi-quantum-well nanopillar arrays (MQW-NPAs) with different lateral sizes. These analyses will be helpful in understanding the optical properties of other microstructured materials.

## Methods

Ten periods of Ge (0.9 nm)/Si (12 nm) multi-quantum-well (MQW) samples were grown by a molecular-beam epitaxy (MBE) system (Riber EVA-32 ) on p-type Si wafers (<100> oriented, 0.01–0.02 Ω cm). The wafer was cleaned by a standard Radio Corporation of America (RCA) method and passivated by HF before loading into the MBE chamber. The deposition rates of Si and Ge were 0.625 and 0.1 Å/s, respectively. The wafer was pretreated at 860 °C for 5 min. After that, a 30-nm-thick Si buffer layer was grown at 700 °C. The first 0.9-nm-thick Ge layer was deposited at 510 °C, and the first 12-nm-thick Si spacer layer was deposited at the temperature ramping from 510 to 560 °C. The other Ge quantum well layers and Si spacer layers were deposited at the same deposition temperature and with the same deposition amount. Finally, a 130-nm-thick Si cap layer was deposited at 560 °C. After the growth, the sample was in situ annealed at 680 °C for 5 min in order to reduce point defects that probably formed at the low growth temperature of 560 °C.

**Fig. 1:**
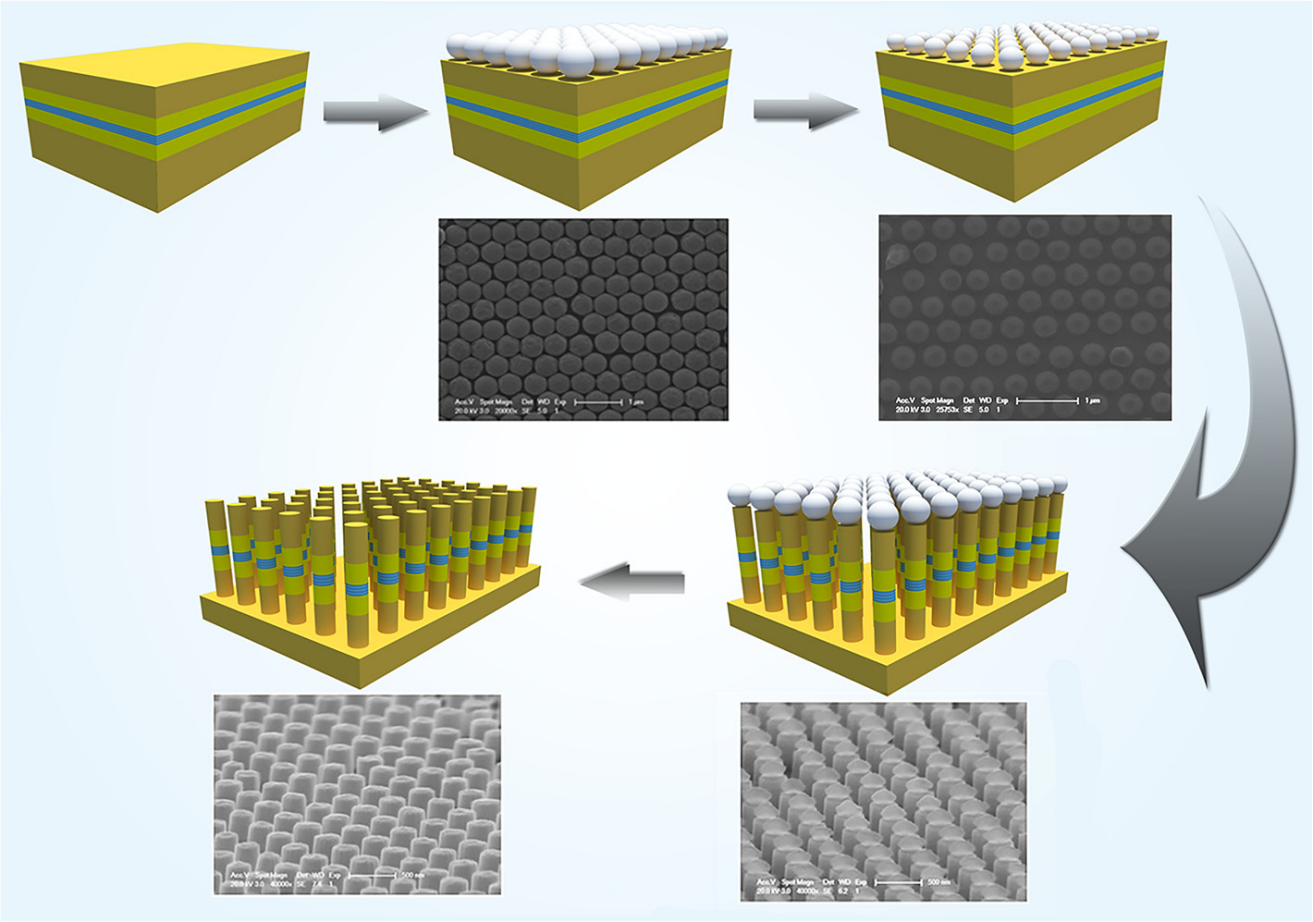
Scheme of the fabrication procedures of GeSi MQW-NPAs

The fabrication procedures of large-area ordered GeSi MQW-NPAs are illustrated in Fig. 1. Firstly, the sample was cleaned by a standard RCA process. After that, a monolayer of 500-nm closed-packed polystyrene nanosphere (PS) arrays was self-assembled onto the substrate; then, the substrate was baked at 80 °C for 3 min to remove the residual deionized water and other impurities. Next, the size of the PS was etched to a required diameter for the nanopatterned mask (~450, 350, and 250 nm) by oxygen plasma etching with a radio-frequency (RF) power of 30 W. After that, the sample was etched to nanopillar array structures with an ion-coupling plasma (ICP) process under the surface protection by PSs. Finally, the remaining PSs were removed via another oxygen plasma-etching process.

Morphologies of GeSi MQW-NPAs were characterized by a field emission scanning electron microscopy (FE-SEM; XL30FEG, PHILIPS, Netherlands) equipped with an energy dispersive X-ray spectrometer (EDS) operating at 20 kV. Before SEM measurements, a 20-nm-thick Au layer was deposited onto the surface in order to get more distinct images. Hemispherical optical reflectance spectra of planar Si bulk and MQW-NPAs were collected in the wavelength range between 300 and 1100 nm (step, 5 nm) via a UV-Vis spectrometer. For PL measurements, the samples were placed in a closed-cycle helium cryostat with a temperature range from 16 K to room temperature, and a 488-nm line of Ar+ laser was used as the excitation source. The PL spectra were recorded by an extended InGaAs detector using the standard lock-in technique. Numerical simulations for the spatial distributions of optical field intensity were performed based on a rigorous coupled-wave analysis (RCWA) method and three-dimensional finite-difference-time-domain (FDTD) method.

## Results and Discussion

**Fig. 2:**
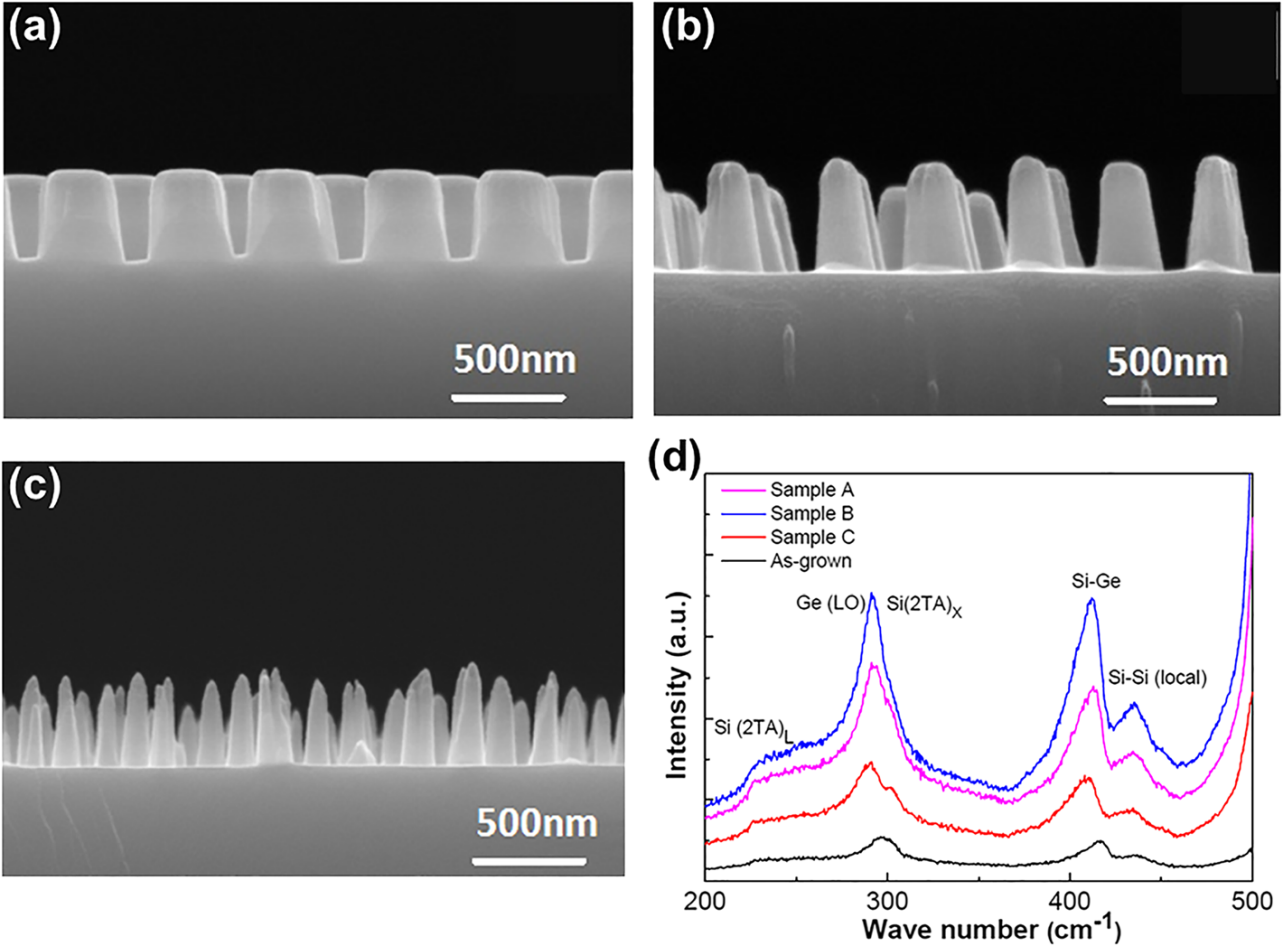
Cross-sectional SEM images of GeSi MQW-NPAs. **a** Sample A. **b** Sample B. **c** Sample C. **d** Raman spectrum of GeSi MQWs and GeSi MQW-NPAs

Figure 2a–c shows cross-sectional SEM images of MQW-NPAs of samples A, B, and C, which correspond to the nanopatterned mask diameters of 450, 350, and 250 nm, respectively. It can be seen that the MQW-NPAs were vertically aligned on the substrates with well periodical structures in a large-scale area. The final geometries of MQW-NPAs are like circular truncated cones, because MQWs and nanosphere masks were etched at the same time. For samples A, B, and C, the top diameters of the nanopillars are ~270, ~120, and ~70 nm, respectively, and the bottom diameters are ~340, ~200, and ~140 nm, respectively. The nanopillar sample heights of all the three samples are about 400 nm. In order to analyze the crystal quality of the samples after ICP etching, Raman measurements were performed. As shown in Fig. 2d, for the as-grown MQW sample, the peaks at 230, 302, 417, and 437 cm^−1^ are assigned to Si(2TA)_L_, Si(2TA)_X_, Si-Ge phonon mode, and Si-Si phonon (local) modes, respectively. For the ICP-etched samples, both peaks of Ge(LO) and Si-Ge mode have a redshift compared to the as-grown MQW sample, which is mainly resulting from the compressive strain relaxation [13]. The Raman results prove that the crystalline quality of the ICP-etched samples was not damaged during the etching process.

**Fig. 3:**
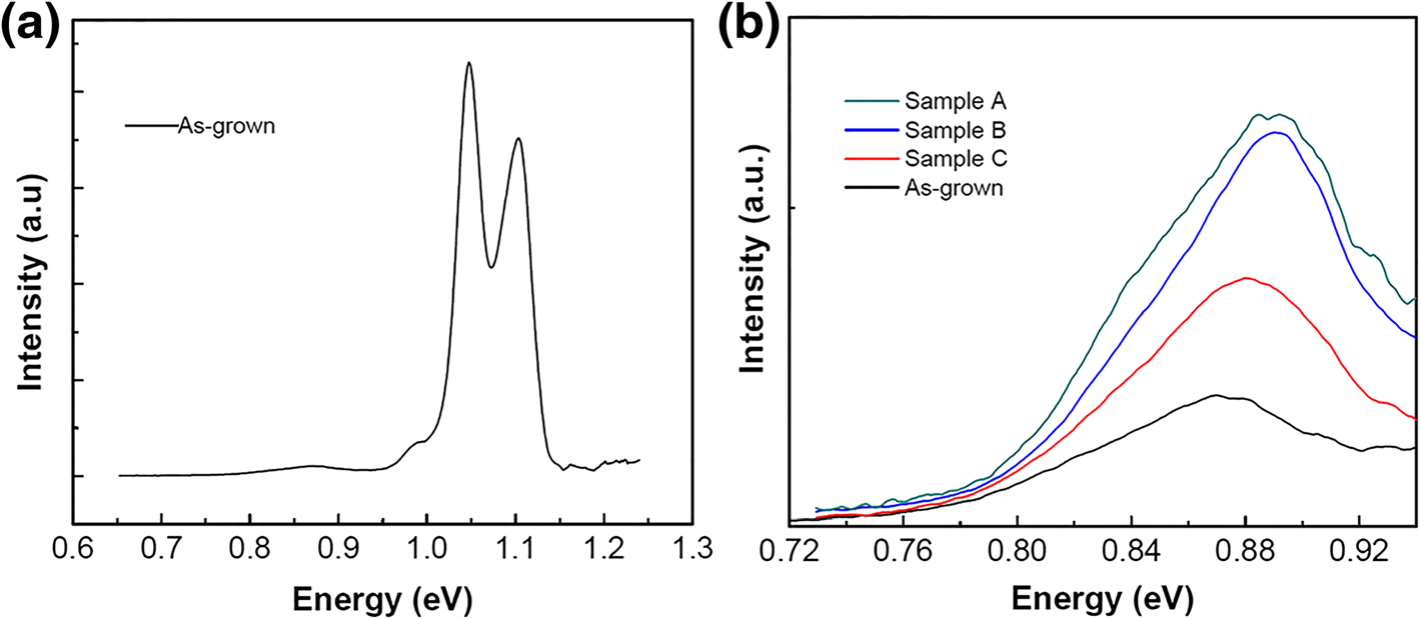
**a** PL spectra of as-grown GeSi MQWs. **b** PL spectra of as-grown GeSi MQWs and GeSi MQW-NPAs in a small scale

Figure 3a shows the PL spectra of the as-grown MQW sample. The measurement was carried out at 16 K at an excitation power of 1 W. Two main peaks located at about 1.1 and 1.05 eV are attributed to transverse optical (TO) phonon-assisted recombination in the Si spacer layer and the doped Si substrate, respectively [16]. The broad weak peak at ~0.88 eV is attributed to the PL of GeSi MQWs. Figure 3b shows the PL spectra in the wavelength range from 0.72 to 0.93 eV. It can be seen that the PL intensity of GeSi MQW-NPA samples increased considerably compared with the as-grown MQW sample. We introduce a PL enhancement factor (PEF), PEF = *I*_NP_/*I*_QW_, to quantitatively describe the PL enhancement, where *I*_NP_ and *I*_QW_ represent the integrated intensities of the PL of the MQW-NPA samples and the as-grown sample, respectively. As shown in Table 1, the sample A has the largest enhancement, with a PEF of 2.61. Sample B and sample C have 2.25 and 1.55 PEF factors, respectively. The PEF increases with nanopillar lateral size. In addition, the peaks of the MQW-NPA samples show a small blueshift compared with the as-grown MQWs.

**Table 1:** PEF, AEF, and EEF of GeSi MQWs and GeSi MQW-NPAs

	PL enhancement factor (PEF)	Absorption enhancement factor (AEF)	Extraction enhancement factor (EEF)
As-grown MQWs	1	1	1
Sample A	2.61	1.55	2.66
Sample B	2.25	1.4	1.02
Sample C	1.55	1.18	0.34

As mentioned above, there are several mechanisms proposed to explain the PL enhancement in the nanopatterned surface sample case. In the following, we will discuss the enhancement in more detail and with quantitative calculations. Firstly, the possibility that the PL enhancement mainly comes from the localization of excitons, i.e., quantum confinement, is ruled out, simply because of the two factors. One is the diameters of the nanopillars largely exceed the exciton Bohr radius of Ge and Si (~10 nm). The second is the MQW-NPA sample with a larger diameter has a large PEF, which is opposite to the attribution of localization of excitons.

**Fig. 4:**
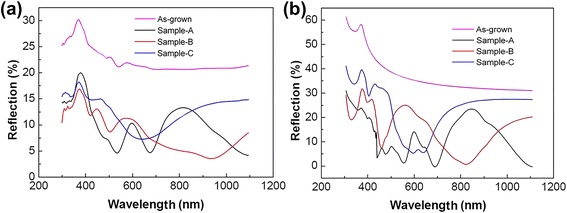
**a** Total hemispherical optical reflectance of GeSi MQWs and GeSi MQW-NPAs. **b** Simulated total hemispherical reflectance of GeSi MQW-NPAs

Normally, a nanopatterned surface can be regarded as an antireflection layer, depending on the surface morphologies and wavelength of incident light. In our case, the large-area ordered GeSi MQW-NPAs can effectively reduce the reflection of incident light, according to the previous results [17–19], which could increase the absorption of incident light and result in PL enhancement. In order to verify the effect on the light trapping, both reflectance spectrum measurements and FDTD simulations were carried out, as shown in Fig. 4. The simulation results are consistent with the experimental results, and the total hemispherical optical reflectance of GeSi MQW-NPA samples was all lower than that of the as-grown GeSi MQWs. Moreover, as shown in both Fig. 4a, b, it is clearly seen that the optical reflectance of the GeSi MQW-NPA samples decreases with increasing lateral size of the nanopillars at the incident light wavelength of 488 nm. Here, we define the absorption enhancement factor (AEF), AEF = *A*_NP_/*A*_QW_, where *A*_NP_ and *A*_QW_ respectively represent the absorptances of the MQW-NPA samples and the as-grown MQW sample at the wavelength of 488 nm, which are calculated by one minus reflectance. As shown in Table 1, sample A has the largest AEF and the AEF increases with increasing size of the nanopillars.

**Fig. 5:**
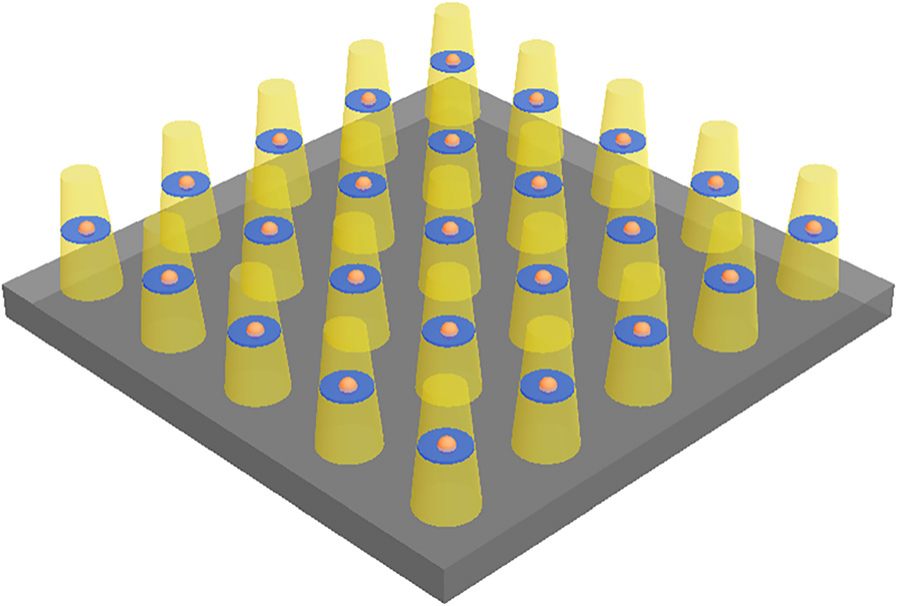
Model of the MQW-NPA samples for FDTD simulations. The *blue plane* is at *z* = 0. The *orange dots* refer to the emission dipoles

As shown in Table 1, the AEF values of the nanopillar samples are smaller than the corresponding values of the PEF, which implies that the increased light absorptance cannot be regarded as the only reason for the PL enhancement of the nanopillar samples. So, we need to find the other reasons for the PL enhancement. It is well known that the photonic crystals could increase the extraction efficiency of lights [20]. Since the nanopillars in MQW-NPA samples are periodically arranged on the surfaces, photonic crystal effect should be taken into account. FDTD simulations were performed at emission wavelength *λ* = 1410 nm (0.88 eV) to further explore the reasons for the PL enhancement. In the simulations, the center of the nanopillar was taken as the coordinate origin. An emission dipole was embedded in the QW plane (*z* = 0), as shown in Fig. 5. The method to calculate the extraction enhancement factor (EEF) can refer to Yue’s work [21]. At first, the extraction efficiency of a single dipole was simulated at different positions. Then, we took the average value on the extraction efficiency. Finally, we define the EEF = *E*_NP_/*E*_QW_, where *E*_NP_ and *E*_QW_ represent the extraction efficiencies of the MQW-NPA samples and the as-grown MQW sample, respectively. As shown in Table 1, the EEF of samples A, B, and C are found to be 2.66, 1.02, and 0.34, respectively.

It is noted that for sample C the EEF value is 0.34, and the AFE value times the EEF value equals 0.40, which is much smaller than the observed PEF value of 1.55. This implies that we need to find another possible reason to explain the PL enhancement for sample C.

As we know, the penetration depths of the 488-nm line of the Ar+ laser in Ge and Si are about 16 and 400 nm, respectively [15, 22]. For the as-grown GeSi MQW sample, most electron-hole (e-h) pairs are generated in the top Si layer and the upper GeSi QW regions. The carriers generated in the top Si layer need to be transported to the QW regions and be captured, then the recombination of the e-h pairs in the QWs results in PL from the QWs. For the GeSi MQW-NPA samples, the effective penetration depth of light becomes larger, which results in two consequences. (1) More e-h pairs are generated at the QW regions and captured directly by QWs without carrier transport loss. (2) More e-h pairs are generated at and captured by the bottom GeSi QWs. These bottom QWs should have a better quality than the upper QWs and thus have a higher PL efficiency since the bottom QWs actually underwent a longer annealing process during the growth [23, 24]. These two consequences lead to an enhancement in PL intensity for the MQW-NPA samples compared to the as-grown GeSi MQW sample.

**Fig. 6:**
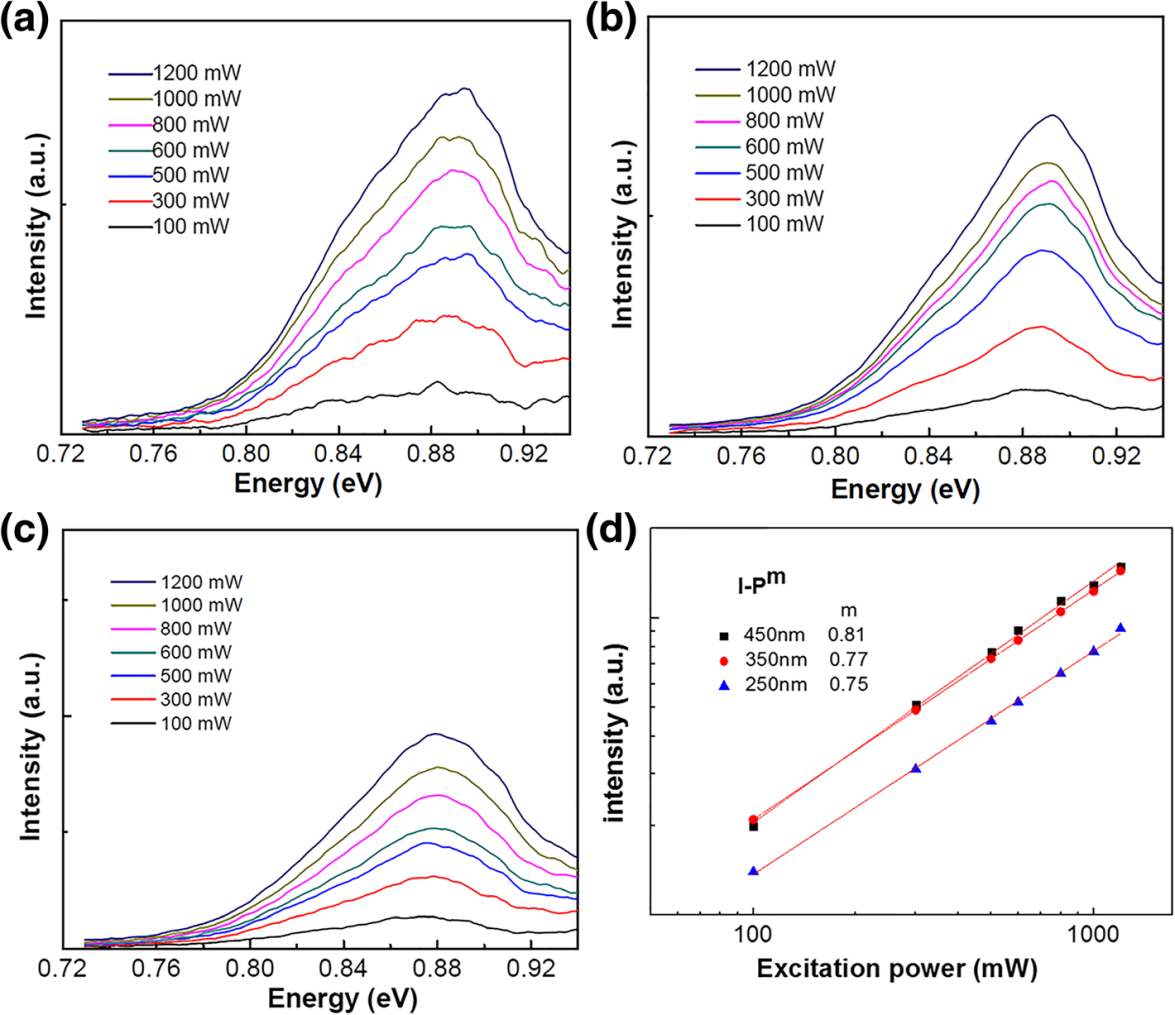
PL spectra of GeSi MQW-NPAs as a function of excitation power at 16 K. **a** Sample A. **b** Sample B. **c** Sample C. **d** Integrated PL intensity of GeSi MQW-NPAs as a function of excitation power

Figure 6a–c shows excitation power-dependent PL spectra of samples A, B, and C, respectively. Measurements were carried out at 16 K. Figure 6d shows the integrated PL intensity as a function of the excitation power (*I* ~ *P*^*m*^) for the three GeSi MQW-NPA samples. The coefficients *m* are found to be 0.75, 0.77, and 0.81 for the samples A, B, and C, respectively. This means the band alignment for the GeSi MQW-NPA samples is type II since the *m* values are between 0.5 and 0.8, which are typical values for type II band alignment for the Ge/Si heterostructures [25]. Besides, for the three samples, with increasing excitation power, the peak linewidth becomes narrower and the peak energy has a blueshift, which is similar to the PL behavior of the GeSi quantum dot system [16]. For the type II band alignment, electrons and holes are resident in Si layers and GeSi quantum-well layers, respectively, being separated spatially. With increasing excitation power, more electrons and holes are generated, which make the band bending more severe in the case with type II band alignment. At the same time, more electrons and holes will make the carriers fill higher energy level states. Both effects: band filling effect and band bending effect, would make the PL peak energy blueshift. In fact, with increasing excitation power, blueshifts in PL spectra have been observed in many GeSi systems with type II band alignment, which are also attributed to those two effects [26–28].

## Conclusions

We successfully fabricate large-area ordered GeSi nanopillar arrays with different lateral sizes (270, 120, and 70 nm) by a cost-effective nanosphere lithography technique. Compared to the as-grown GeSi MQW sample, the GeSi MQW-NPA samples with different lateral sizes show different enhancements in PL intensity. The GeSi MQW-NPA sample with the largest nanopillar size shows the largest enhancement, which is as high as 2.61. The enhancements are attributed to three factors, antireflection of nanopillar structures, enhanced extraction efficiency, and more contribution to the PL from bottom GeSi QWs. Three factors are analyzed quantitatively or qualitatively, and each plays an important role for the PL enhancement. Those analyses can help understand the optical properties of the MQWs of surface microstructures as well as design surface microstructures in order to improve their optical properties.

## Abbreviations

AEF: absorption enhancement factor
EEF: emission enhancement factor
FDTD: finite-difference-time-domain
ICP: ion-coupling plasma
MBE: molecular-beam epitaxy
MQW-NPAs: multi-quantum-well nanopillar arrays
MQWs: multi-quantum wells
PEF: PL enhancement factor
PL: photoluminescence
